# The Autoantibodies against Tumor-Associated Antigens as Potential Blood-Based Biomarkers in Thyroid Neoplasia: Rationales, Opportunities and Challenges

**DOI:** 10.3390/biomedicines10020468

**Published:** 2022-02-17

**Authors:** Pavel V. Belousov

**Affiliations:** 1 National Center for Personalized Medicine of Endocrine Diseases, National Medical Research Center for Endocrinology, Ministry of Health of the Russian Federation, 117036 Moscow, Russia; belousov.pavel@endocrincentr.ru or belousp@gmail.com; 2 Center for Precision Genome Editing and Genetic Technologies for Biomedicine, Engelhardt Institute of Molecular Biology, Russian Academy of Sciences, 119991 Moscow, Russia

**Keywords:** anti-tumor immunity, autoantibody, biomarker, diagnostics, follicular-patterned thyroid tumor, indeterminate cytology, thyroid cancer, thyroid neoplasm, thyroid nodule, tumor-associated antigen

## Abstract

The Autoantibodies targeting Tumor-Associated Antigens (TAA-AAbs) emerge as a result of a variety of tumor-related immunogenic stimuli and may be regarded as the eyewitnesses to the anti-tumor immune response. TAA-AAbs may be readily detected in peripheral blood to unveil the presence of a particular TAA-expressing tumor, and a fair number of TAAs eliciting the tumor-associated autoantibody response have been identified. The potential of TAA-AAbs as tumor biomarkers has been extensively studied in many human malignancies with a major influence on public health; however, tumors of the endocrine system, and, in particular, the well-differentiated follicular cell-derived thyroid neoplasms, remain understudied in this context. This review provides a detailed perspective on and legitimate rationales for the potential use of TAA-AAbs in thyroid neoplasia, with particular reference to the already established diagnostic implications of the TAA-AAbs in human cancer, to the windows for improvement and diagnostic niches in the current workup strategies in nodular thyroid disease and differentiated thyroid cancer that TAA-AAbs may successfully occupy, as well as to the proof-of-concept studies demonstrating the usefulness of TAA-AAbs in thyroid oncology, particularly for the pre-surgical discrimination between tumors of different malignant potential in the context of the indeterminate results of the fine-needle aspiration cytology.

## 1. Introduction

The incidence of nodular thyroid disease (NTD) and thyroid cancer have grown rapidly in the last few decades, largely as a result of the widespread use of thyroid ultrasonography (US) and consequently frequent incidental detection of clinically silent nodules (reviewed in [1]). The management of patients with NTD issues a great challenge for public health and requires careful stratification of patients according to the risk of malignancy (reviewed in [2,3]). Apart from non-neoplastic lesions constituting the vast majority of thyroid nodules, the remaining neoplastic processes are predominantly represented by the well-differentiated follicular cell-derived tumors. These are further classified into papillary thyroid carcinoma (PTC) and variants thereof, follicular and Hürthle cell carcinomas (FTC and HCC, respectively), and their benign counterparts follicular and Hürthle cell adenomas (FTA and HCA, respectively); additional borderline lesions include the non-invasive follicular thyroid neoplasm with papillary-like nuclear features (NIFTP), a distinct indolent entity recently partitioned from the encapsulated follicular variant of PTC (FV-PTC), as well as follicular and well-differentiated tumors of uncertain malignant potential (FT-UMP and WDT-UMP, respectively) [4]. Other entities such as the medullary (MTC), poorly differentiated (PDTC), and anaplastic (ATC) thyroid carcinomas have a very low prevalence in the general population of patients with NTD and thus are out of the primary scope of this review; still, I refer to these entities in a few particular contexts (see Section 5).

The current strategies of management of patients with NTD and differentiated thyroid cancer (DTC) (comprising all follicular cell-derived malignant neoplasms listed above except PDTC and ATC) [2,3,5,6,7,8,9], although being successfully implicated in routine clinical practice, still suffer from several drawbacks and pitfalls, thereby creating highly relevant windows for improvement (see Section 4.2). In particular, whereas the majority of PTC variants (with a notable exception of FV-PTC) are usually confidently diagnosed using the fine-needle aspiration (FNA) cytology owing to the abundant presence of characteristic cytomorphological and architectural features (e.g., the PTC nuclear features, true papillae, and psammoma bodies), the preoperative distinguishing between the remaining entities (that may be collectively referred to as follicular-patterned tumors (FPT)), especially concerning the malignant potential of a lesion, remains a significant challenge [7]. This clinical demand, along with several other contexts discussed in Section 4.2, provides a number of highly relevant diagnostic niches for the various types of biomarkers.

One particular type of tumor markers that was extensively implicated in human cancer but remains understudied in thyroid neoplasia is circulating autoantibodies (AAbs) against tumor-associated antigens (TAAs) (see Section 2, reviewed in [10,11,12]). A variety of experimental techniques for a systematic survey of TAAs’ and TAA-AAbs’ repertoires have been successfully implemented for the identification of a large number of TAAs; yet, the technical aspects of TAAs’ identification are out of scope of the present review, and I refer readers to recent comprehensive reviews in the field [13,14,15]. In this review, I provide a detailed perspective on the potential diagnostic use of TAA-AAbs in thyroid neoplasia, with particular reference to the already established diagnostic implications of the TAA-AAbs in human cancer (Section 3), as well as to the current workup strategies in thyroid oncology (Section 4.1), specifically highlighting potential windows for improvement thereof (Section 4.2). I further discuss the published research on thyroid neoplasia-associated TAAs/TAA-AAbs (Section 5) and the problems and limitations associated with the potential use of TAA-AAbs in various specific clinical contexts (Section 6) in order to ultimately provide legitimate rationales for the diagnostic use of TAA-AAbs in thyroid oncology and propose the specific clinically-relevant diagnostic niches that TAA-AAbs may successfully occupy.

## 2. The Basics of TAAs and TAA-AAbs in Human Cancer

TAAs are wild-type self-proteins that are normally expressed either ubiquitously or in a tissue or ontogenic stage-specific manner, albeit being capable of triggering the immune response upon expression by tumor cells. The immune responses against TAAs result from a tumor-related breakdown of the peripheral immunological tolerance and are mediated by ab initio autoreactive lymphocytes that have escaped from the central tolerance. For example, such lymphocytes may normally stay «ignorant» to their cognate antigens either because the affinity of the antigen receptor is too low to elicit an antigen-specific response or because of the low density of such an antigen in healthy tissues, its restricted expression in the immunologically privileged tissue(s), or both (reviewed in [16]). These ignorant lymphocytes are neither clonally deleted nor rendered anergic and may thus be fully activated upon overexpression of a normally low-abundant TAA or aberrant expression of a TAA normally expressed behind a specialized barrier (e.g., the cancer-testis (reviewed in [17,18,19]) and onconeural (see Section 3.4) antigens). Other relevant immunogenic stimuli include altered protein structure, aberrant post-translational modifications, the aberrant release of intracellular antigens, etc. (reviewed in [10]). In terms of the B-cell branch of the adaptive immunity, such an immune response ultimately results in the production of the TAA-AAbs that may be detected in circulation to unveil the presence of a particular TAA-expressing tumor. As compared with the traditional tumor markers and the diagnostic targets of the liquid biopsy (e.g., circulating tumor cells, cell-free tumor DNA/RNA, etc.) (reviewed in [20,21]), the TAA-AAbs are characterized by a set of unique properties rendering them particularly suitable for some of the diagnostic applications but significantly limiting their use in others [10,11,12].

Many protein tumor markers that may be highly sensitive and specific in the context of a tissue-based diagnostics (e.g., the immunohistochemical (IHC) analysis) do not enter the circulation and, in order to be successfully used for diagnostic purposes, require invasive procedures (e.g., biopsy or diagnostic surgery) with associated risks, patient emotional distress and health care costs. In contrast, TAA-AAbs are soluble circulating proteins and may be easily determined in a minute amount of serum or plasma, using either conventional robust methods (such as ELISA analysis) or more state-of-the-art multiplexed assays such as customized Luminex^®^ panels.

Furthermore, the antibodies are highly stable plasma proteins with long half-lives in circulation due to limited proteolysis and clearance. This implies that the requirements for the preparation for the test (e.g., fasting or physical activity), as well as those for sample handling and the short-term storage, are relatively relaxed to virtually absent; however, the same issue somewhat compromises the use of TAA-AAbs for disease monitoring in the majority of cancers, with the notable exception of slow-growing malignancies, with DTC being a good representative thereof (see Section 4.2).

Concerning the traditional circulating tumor markers and the targets of the liquid biopsy, the circulating levels thereof are directly proportional to the tumor load, thus limiting their use for the early cancer diagnosis. By contrast, even a subtle amount of a highly immunogenic TAA expressed by a microscopic tumor lesion may be sufficient to trigger a strong and easily detectable antibody response [22].

Finally, the autoantibodies against *bona fide* TAAs usually demonstrate very high diagnostic specificity (DSp) in the detection of malignancy (typically 90–95% or higher), whereas their diagnostic sensitivity (DSn) is usually low-to-moderate (typically 10–25% for a single TAA-AAb) [12]. It implicates the TAA-AAbs as the perfect candidates for the «rule in» tests, whereas their use for «rule out» diagnostics usually requires a combination of TAA-AAbs against several TAAs in a single diagnostic panel, also referred to as autoantibody signature (such as in EarlyCDT^®^-Lung test, see Section 3.2), concurrent use of TAA-AAbs with other types of biomarkers or diagnostic approaches, or use of the benign tumor-associated TAA-AAbs that disappear upon progression to malignancy and thus may be used for implicit ruling out of malignancy via confident ruling in the benign nature of a tumor (see Section 6). The incorporation of highly specific TAA-AAbs in the state-of-the-art patients’ workup may be particularly beneficial in those clinical scenarios where conventional diagnostics is capable of efficiently ruling out malignant disease in the test-negative patients, while the relatively low diagnostic specificity results in unnecessary invasive procedures in a significant proportion of the test-positive patients who are actually free of cancer (e.g., the molecular test-positive thyroid nodules with «indeterminate» results of the FNA cytology (see Section 4.2)).

## 3. The Diagnostic Implications of TAA-AAbs in Human Tumors

In modern oncology, various types of biomarkers may be used in multiple clinical settings, including estimating the risk of cancer in the future, early diagnosis or screening, the differential diagnosis between benign and malignant lesions, or between different types of cancer (e.g., between tumors of different tissue origin or histological type), determining whether a patient may benefit from a particular treatment (e.g., the analysis of expression of a druggable molecular target), determining the prognosis, or monitoring for local or distant relapse after initial treatment.

### 3.1. Early Diagnosis/Screening

Historically, the majority of TAA-AAbs were primarily identified and studied with the intention of achieving early cancer detection, mainly due to a remarkable ability of TAA-AAbs to signal the presence of cancer long before the life-threatening tumor becomes clinically evident [23]. For example, circulating TAA-AAbs against p53 (p53-AAbs) may be detected long (up to 11 years) before the development of liver angiosarcoma associated with the chronic occupational exposure to vinyl chloride [24]; similarly, p53-AAbs were detected 17–47 months prior to the clinical manifestation of lung cancer in uranium workers [25]. Importantly, such a pattern is probably a rule rather than an exception. For example, Jett et al. [26] evaluated the detection lead time for a panel of 14 TAAs, including seven antigens from the EarlyCDT^®^-Lung panel (see Section 3.2), in 142 postmenopausal women recruited in The United Kingdom Collaborative Trial of Ovarian Cancer Screening (UKCTOCS) who were ultimately diagnosed with lung cancer during the 10-year follow-up. The median detection lead time of TAA-AAbs across a panel of 14 TAAs was 4.1 years (0.1 to 9.0 range).

Interestingly, once a TAA is a soluble protein capable of being secreted by or shed or released from the tumor cells, the TAA along with the cognate TAA-AAbs may complement each other in terms of diagnostic performance at the different stages of tumor progression. For example, using the prediagnostic breast cancer plasma samples collected as a part of the Women’s Health Initiative (WHI) observational study, Ladd et al. [27] demonstrated that the circulating levels of PKM2, the oncogenic isoform of the pyruvate kinase, the key mediator of the Warburg effect in malignant tumors [28], and a well-established tumor marker Tumor M2-PK [29,30,31,32], are particularly high in the blood plasma samples collected within the 150 days prior to the diagnosis of the breast carcinoma, but not in samples collected further from diagnosis. By contrast, TAA-AAbs against PKM2 (PKM2-AAbs) demonstrate the opposite trend, with the highest level of PKM2-AAbs being observed in samples collected further from diagnosis. The likely explanation of such a pattern is the formation of the circulating immune complexes (CICs) incorporating particular TAAs and TAA-AAbs. This process may interfere with the immunochemical detection of both analytes, with the apparent level of each of them depending upon the CICs’ stoichiometry at a particular time point. The AAbs-mediated interference in immunochemical assays was also described for circulating tumor markers CA-15.3 [33], CA-125 [34,35], and, most importantly, for thyroglobulin (TG), a tumor marker of DTC recurrence following primary treatment (see Section 4.2). In these cases, the TAA-AAbs may not only indicate that the results of a tumor marker’s measurements may not be reliable but also provide additional diagnostic information, such as in DTC patients with rising anti-thyroglobulin antibodies (TG-AAbs) (see Section 4.2).

Yet another feature of TAA-AAbs that also relates to the use of TAA-AAbs as biomarkers for the early cancer diagnosis is their remarkably high diagnostic specificity (DSp) [12]. In the setting of a population screening, due to a low overall prevalence of cancer, the negative predictive value (NPV) of a screening test (i.e., the percentage of true negative results among individuals who are tested negative) may be very high despite a low-to-moderate DSn value. At the same time, the achievement of minimally acceptable positive predictive value (PPV), i.e., the percentage of true positive results among individuals who are tested positive, requires very high DSp values, such as those typically demonstrated by autoantibodies against *bona fide* TAAs.

### 3.2. The Differential Diagnosis

Once the cancer is suspected, a common diagnostic challenge is distinguishing «true» cancer from a variety of benign conditions demonstrating the overlapping clinical, radiological and even cytomorphological features but requiring less aggressive management (e.g., conservative treatment, organ and tissue-sparing surgery, etc.) or no treatment at all (see Section 4.1). As compared with the early diagnosis, fewer studies specifically addressed the issue of the differential diagnosis between benign and malignant lesions. Nevertheless, the published data suggest that TAA-AAbs may be successfully used in this diagnostic context as well.

One such context is discriminating between prostate cancer (PC) and benign prostatic hyperplasia (BPH), which may not be reliably accomplished using the measurement of serum prostate-specific antigen (PSA), resulting in many unnecessary biopsies [36]. For example, using iterative biopanning of a phage-display library derived from prostate cancer tissues, Wang et al. [37] used phage protein microarrays to end up with a 22-features phage-peptide detector capable of discriminating between PC and BPH with the area under receiver-operating curve (ROC AUC) values 0.93–0.94. Such excellent diagnostic performance was particularly evident in groups of patients with PSA levels of 2.5–10 ng/mL, where the discriminatory capacity of the PSA level was close to zero (ROC AUC 0.5–0.55). In another study, O’Rourke et al. [38] used a low-density native antigen reverse capture microarray comprising monoclonal antibodies against 27 pre-selected TAAs to ultimately select five TAAs (PARK7, CALD1, TARDBP, TLN1, and PSIP1) capable of discriminating between the PSA level-matched groups of patients with PC and BPH with ROC AUC 0.95 compared with that of PSA 0.5 (i.e., complete lack of discriminative power).

Several other TAAs and TAA-AAbs signatures were further implicated in the differential diagnosis between PC and BPH [39,40], breast cancer and benign breast disease [41], benign and malignant salivary gland tumors [42], ovarian cancer and benign ovarian tumors [43], etc. However, the most successful implication of TAA-AAbs as a tool for differential diagnosis is probably represented by the EarlyCDT^®^-Lung test [44,45,46,47,48,49] commercially available in the USA via the Clinical Laboratory Improvement Amendments (CLIA), the test comprising a 7-TAA panel (p53, CTAG1B/NY-ESO-1, CAGE, GBU4-5, HuD/ELAVL4, MAGEA4, and SOX2) and currently being positioned as an adjunctive test to the low-dose screening computed tomography (CT). The values of DSp and DSn and the diagnostic accuracy for all stages or types of lung cancer across all studied cohorts were 92%, 41%, and 93%, respectively [44,45,46,47,48,49], and these values may be further adjusted for particular applications using different criteria for test positivity [50]. The EarlyCDT^®^-Lung panel was validated in a number of independent cohorts [44,45,46,47,48,49], and the administration of this test to patients with incidentally discovered lung nodules who are at intermediate risk of lung cancer was demonstrated to improve patients’ outcomes in a cost-effective manner [51]. These encouraging data are particularly relevant to the topic of the present review, provided that the problem of discrimination between benign and malignant lesions is one of the most critical issues in thyroid oncology (see Section 4.2).

### 3.3. Prognostication

Since the TAA-AAbs represent the direct indicators of the ongoing anti-tumor immune response, one may suggest that their presence reflects a degree of the immune control of a tumor, thereby providing a rationale for their use as indicators of a favorable prognosis [12,13,52,53]. However, the majority of TAAs are intracellular proteins and, apart from rare scenarios described in occasional reports demonstrating the ability of TAA-AAbs to cross the cell membrane [54,55], they are usually regarded as silent witnesses of the anti-tumor immune response rather than its direct effectors. In such a way, the prognostic role of the TAA-AAbs of a particular specificity is probably determined by a balance of the cell-intrinsic role of cognate TAA in tumor progression and the degree to which the presence of these TAA-AAbs parallels the activation of the fully functional effectors of the anti-tumor immunity (e.g., CD8+ cytotoxic T-cells). Hence, the presence of the particular TAA-AAbs may indicate either favorable or poor prognosis or may have no prognostic significance at all. For example, using high-density protein microarrays, Gnjatic et al. [56] identified a number of highly prognostically discriminative TAA-AAbs signatures associated with either favorable (ANXA2 + FAM13B + FER + ZIM2 in ovarian cancer and GAS2 + HERPUD1 + TMOD1 + TMSB10 in pancreatic cancer) or poor (ERRFI1 + PHLDB1 + TRH + TRUB1 in ovarian cancer and CRYBB2 + NR2E3 + PTPRA + ZNF695 for pancreatic cancer) outcomes. It is of particular interest that even TAA-AAbs of the same antigenic specificity may demonstrate the opposite prognostic significance. For example, the presence of the p53-AAbs (see Section 3.1) is a favorable prognostic sign in ovarian cancer [57] and a poor prognostic indicator in breast [58] and lung [59] cancer; however, both directions of prognostic influence were demonstrated in colorectal cancer [60,61]. Furthermore, even if a particular TAA contains the extracellular domain or is capable of high-affinity binding to the extracellular aspect of the plasma membrane, thereby allowing the TAA-AAbs of the cognate specificity to exert their effector functions (e.g., via blocking of receptor-ligand interaction or triggering the antibody-dependent cellular cytotoxicity or complement-mediated cell lysis), both protective and tumor-promoting effects of such TAA-AAbs have been reported [62,63,64,65]. To sum up, the TAA-AAbs may be successfully used for prognostication purposes, although the presence and direction of their prognostic influence highly depend on a particular TAA and a clinical context in a rather unpredictable way; thus, this issue needs to be specifically addressed in each particular case.

### 3.4. The Diagnostics of The Paraneoplastic Autoimmune Disorders (PADs)

Yet another interesting clinical context for the use of TAA-AAbs is the diagnosis of paraneoplastic autoimmune disorders (PADs) (reviewed in [66]). Given that TAAs are, by definition, normal self-proteins (see Section 2), the anti-TAA immune response may result in the autoimmune damage of normal tissues expressing particularly high levels of such a TAA. The PADs’ clinical phenotypes significantly overlap with various benign conditions, whereas their clinical manifestation may significantly precede cancer diagnosis [67,68,69], in line with the appearance of TAA-AAbs long before clinical manifestation of malignancy (see Section 3.1). Hence, in the case of suspected PAD, the TAA-AAbs recognizing such TAAs may be successfully used to verify the paraneoplastic nature of a particular disorder and, in some cases, suggest the localization, tissue lineage, or both of the underlying tumor [70,71,72,73,74]. Even more importantly, many onconeural TAA-AAbs are also encountered in a significant proportion of cancer patients not suffering from any type of PAD. For example, low-titer anti-Hu antibodies recognizing the ELAV (embryonic lethal abnormal vision) family of onconeural antigens are detected in 10–20% of the small-cell lung cancer patients not suffering from the anti-Hu paraneoplastic encephalitis [75,76,77,78], whereas TAA-AAbs against cancer-retina antigen recoverin (RCVRN) are detected in 15–20% of lung cancer patients not suffering from the cancer-associated retinopathy [79]. Hence, such TAA-AAbs may be successfully used for various diagnostic purposes in general oncology apart from the clinical context of the cancer-associated autoimmunity, as exemplified by HuD/ELAVL4 antigen representing one of seven TAAs in the EarlyCDT^®^-Lung panel (see Section 3.2).

## 4. The Clinical Context and Potential Diagnostic Niches for the Autoantibody Biomarkers in Thyroid Neoplasia

**Figure 1:**
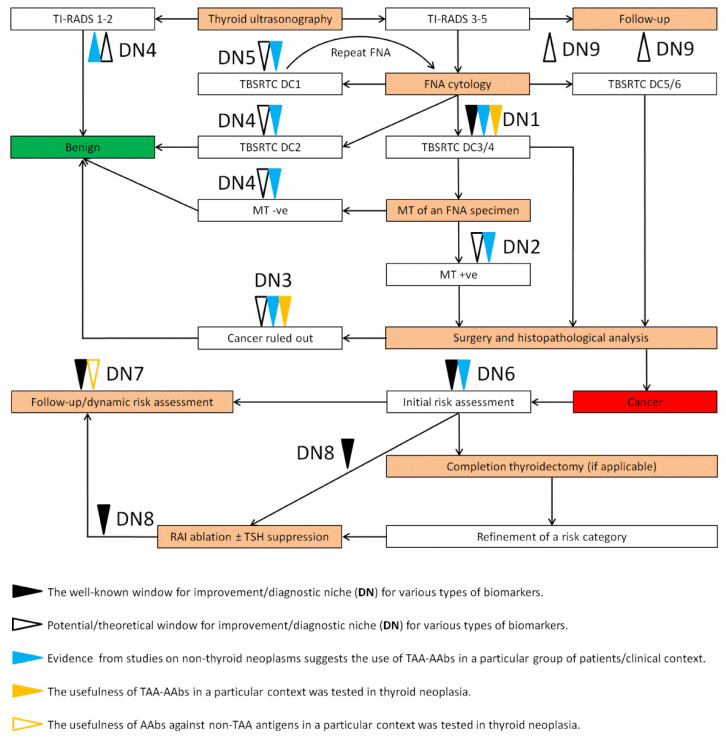
A simplified workflow of the management of patients with NTD and DTC [2,3,5,6,7,8,9] (see Section 4.1 for details) along with potential windows for improvement and diagnostic niches (DNs) that various types of biomarkers, including TAA-AAbs, may potentially occupy (arrowheads, see Section 4.2 for details). The type and strength of evidence suggesting the use of TAA-AAbs in particular groups of patients and clinical contexts are represented by the number and color of arrowheads as deciphered in the figure legend (bottom panel). The text boxes representing diagnostic and treatment procedures are colored in light orange. DN—Diagnostic Niche; FNA—Fine-Needle Aspiration; MT—Molecular Test; RAI—Radioactive Iodine; TAA-AAbs—Autoantibodies against Tumor-Associated Antigens; TBSRTC DC—Diagnostic Category (DC) according to The Bethesda System for Reporting Thyroid Cytopathology (TBSRTC); TI-RADS—Thyroid Imaging Reporting & Data System; TSH—Thyroid-Stimulating Hormone.

The current workup protocols in thyroid oncology incorporate a variety of diagnostic procedures to provide cost-effective management for each patient with symptomatic or incidentally discovered thyroid nodules [2,3,5,6,7,8,9] (Figure 1). The establishment of the Thyroid Imaging and Reporting & Data System (TI-RADS) incorporating various ultrasonographic (US) characteristics of a thyroid nodule to assign it to a particular risk group greatly improved endocrine practice and allowed the risk-based decisions to be made about whether to proceed to the US-guided fine-needle aspiration (FNA) biopsy, schedule the US follow-up, or confidently ascertain the benign nature of the nodule [5,6].

### 4.1. The Basics of the Current Management of Patients with Nodular Thyroid Disease

When the US-guided FNA biopsy is indicated, the cytological evaluation of the FNA specimen is performed to further assign the nodule to one of six diagnostic categories (DCs) according to The Bethesda System for Reporting Thyroid Cytopathology (TBSRTC) [7]. Depending on the TBSRTC DC of the FNA cytology (Figure 1), the patient may be assured of the benign nature of his nodule(s) (TBSRTC DC2/«benign»), referred for curative surgery (TBSRTC DC5/«suspicious for malignancy» and TBSRTC DC6/«malignant»), diagnostic lobectomy (TBSRTC DC3/«atypia of undetermined significance or follicular lesion of undetermined significance», and TBSRTC DC4/ «follicular neoplasm/suspicious for follicular neoplasm»), repeating of FNA (TBSRTC DC1/ «nondiagnostic/unsatisfactory» and TBSRTC DC3), or adjunctive molecular testing to further refine the malignant potential of a tumor (TBSRTC DC3 and TBSRTC DC4, see Section 4.2).

Once the surgery is indicated and performed, the pathological examination of surgical specimens allows the establishment of a final pathological diagnosis and accurate staging once the cancer is discovered or verified. Depending on the particular risk group assigned, a careful follow-up may be scheduled, or a patient may be referred for a completion thyroidectomy (in case the initial surgical procedure was a diagnostic lobectomy) or radioactive (^131^I) iodine (RAI) therapy to ablate the thyroid remnant after a near-total thyroidectomy and possible distant metastases (Figure 1). The further follow-up of DTC is primarily based on the regular serum thyroglobulin (TG) measurement and neck US with FNA biopsy once a lesion suspicious for a locoregional recurrence is discovered [8,9].

### 4.2. Potential Diagnostic Niches for TAA-AAbs Biomarkers in Thyroid Neoplasia

Probably the most critical issue that is still far from being completely resolved pertains to the «indeterminate» categories of the FNA cytology, particularly TBSRTC DC3 and TBSRTC DC4, collectively constituting approximately one quarter of all nodules undergoing FNA biopsy [80] with a trend towards a further increase of their prevalence [81]. Given that a significant proportion of TBSRTC DC3 and, in particular, TBSRTC DC4 nodules are proven to be predominantly or purely follicular-patterned tumors (FPT) of varying malignant potential with no or moderate cytological and architectural atypia to be uncovered using FNA cytology [82,83,84,85,86], at this point the only definitive way to establish an accurate diagnosis is a diagnostic lobectomy followed by pathological examination of the resected specimens, allowing the presence and extent of microscopic tumor invasion into and through its own capsule, surrounding thyroid parenchyma, and blood vessels to be assessed. Given the low-to-moderate risk of malignancy (10–40%) in TBSRTC DC3/DC4 nodules [7], a biomarker capable of providing a reliable distinction between benign and malignant processes in these lesions would be of great value (Figure 1-DN1, see also Section 5).

The state-of-the-art molecular testing (reviewed in [87]), in particular, the ThyroSeq^®^ v3 platform [88,89,90], proved to be very useful in TBSRTC DC3 and TBSRTC DC4 nodules. With the unprecedentedly high DSn (pooled value 99% [95% CI 90–100%] reported in a recent meta-analysis [91]), ThyroSeq^®^ v3 is capable of accurately ruling out malignancy in approximately three-quarters of TBSRTC DC3 and TBSRTC DC4 nodules [89]. However, the most frequent genetic alterations characteristic of FPT (e.g., *HRAS*, *KRAS*, and *NRAS* mutations) demonstrate comparable prevalence in benign, borderline, and malignant FPT [92,93,94,95,96,97,98], resulting in only moderate DSp of this test (pooled value 64% [95% CI 32–87%] [91]), with other diagnostic tests available on the market demonstrating even lower DSp values (Afirma Gene Expression Classifier (GEC) [99]: 19% [95% CI 15–24%]; Afirma Gene Sequencing Classifier (GSC) [100,101]: 51% [95% CI 33–69%] [91]). Thus, a highly specific biomarker, such as TAA-AAbs panels, capable of further risk stratification in molecular test-positive nodules, would be of considerable value for these patients (Figure 1-DN2).

The overlapping morphological phenotypes and molecular signatures in FPT of various malignant potential create yet another peculiar niche for the use of TAA-AAbs and other types of biomarkers. In minimally invasive follicular-patterned carcinomas, due to the practical inability to completely examine a tumor capsule in all three dimensions with sufficient space resolution (at least in the setting of the routine pathological workup), subtle foci of invasive growth may reside away from the material sectioned for microscopy, thereby precluding cancer diagnosis. It is not uncommon for such foci to be revealed upon deeper sectioning of archival tissue blocks in FPT primarily diagnosed as FTA/HCA or NIFTP, and distant metastases from such primarily misdiagnosed tumors have been reported [102,103,104,105,106]. Given the limitations of molecular testing in the FPT described above, which are essentially the same in the setting of final pathological analysis, other types of biomarkers (including TAA-AAbs) may be useful for the selection of those patients who might benefit from the aggressive pathological workup to uncover the minimally invasive malignancy in a tumor appearing benign at primary pathological examination (Figure 1-DN3, see also Section 5.1). It is of note that, due to the overall low prevalence of such cases among tumors initially classified as benign, the typical diagnostic performance of TAA-AAbs (i.e., high DSp and low-to-moderate DSn) may be particularly suitable in this context, allowing an acceptable PPV to be achieved while maintaining high NPV, the scenario somewhat similar to population screening (see Section 3.1). Furthermore, the same considerations may also be applied to other groups of patients where the prevalence of cancer is low albeit non-zero (Figure 1-DN4/DN5), although the clinical potential of TAA-AAbs in these patients is not self-evident.

Following surgical treatment, multiple clinical and pathological parameters (i.e., patient’s age, size of a tumor, histological variant and high-grade histological features, vascular invasion, gross extrathyroidal extension, completeness of tumor resection, regional lymph node involvement, and distant metastases) are to be considered for the initial post-surgical risk assessment (Figure 1). Although these prognostic factors and their combinations, including various scoring systems, were extensively implemented in routine DTC risk assessment [8,9], they are plagued with many limitations and uncertainties, prompting the development of various types of biomarkers intended for a more accurate and personalized prediction of DTC behavior (reviewed in [107,108]). In non-thyroid human cancers, a number of prognostic TAA-AAbs signatures were successfully identified (see Section 3.3), providing a rationale for the identification and use of such biomarkers for DTC prognostication as well (Figure 1-DN6).

Following optional ^131^I RAI ablation and TSH suppression therapy for high-risk DTC, the regular serum thyroglobulin (TG) measurement and neck US represent the cornerstones of the follow-up (Figure 1) [8,9,109]. TG is produced both by normal and neoplastic thyrocytes and is thus a marker of the presence of differentiated thyroid tissue in the organism. However, in patients with low-risk DTC treated with thyroid lobectomy only, the serum TG is of limited value for predicting or detecting disease recurrence [110,111]. Furthermore, the anti-TG autoantibodies (TG-AAbs) occurring in 10–30% of both healthy individuals and patients with various thyroid neoplasms interfere with the immunochemical TG assays (reviewed in [112]). In the presence of TG-AAbs, the results of TG measurement may not be reliably interpreted given that, depending on the particular TG assay used, both false-negative and false-positive results may emerge. Interestingly, the TG-AAbs gradually decline following the cognate antigen clearance and reappear upon the re-growth of the DTC tissue, so the rising, stable, or newly appearing TG-AAbs in patients following DTC treatment may indicate biochemically incomplete responses to initial treatment or cancer relapse [8,112,113]. However, many issues concerning the use of TG-AAbs as a biomarker for disease monitoring are still to be addressed. In particular, whereas the decreasing of TG-AAbs > 50% or below the functional threshold of the assay are near-absolutely predictive of the absence of disease persistence or recurrence, less than a quarter of patients with stable or rising TG-AAbs following DTC treatment ultimately demonstrated structurally incomplete responses or recurrences during at least one year of follow-up [114,115]. Furthermore, in patients treated with thyroid lobectomy only, the usefulness of TG-AAbs in disease monitoring or prognostication is rather questionable [111]. Thus, adjunctive biomarkers, including TAA-AAbs that specifically detect the presence of cancer rather than differentiated thyroid tissue, are needed in the contexts of DTC monitoring, post-treatment prognostication, and dynamic risk assessment as well (Figure 1-DN7). It is of special note that the comparable kinetics of IgG clearance and reoccurrence (as evidenced by the successful use of TG-AAbs in DTC monitoring) and that of DTC growth provide a unique window of opportunity for TAA-AAbs against *bona fide* TAAs in the setting of disease monitoring in this particular type of human cancer.

## 5. The Tumor-Associated Antigens (TAAs) and their Cognate Autoantibodies in Thyroid Neoplasia

As compared with other common human cancers [12], the repertoire of TAAs and the diagnostic potential of their cognate TAA-AAbs in thyroid tumors are understudied. Intriguingly, at the same time, thyroid cancer is the only human malignancy in which the measurement of a particular autoantibody biomarker is incorporated into the routine patients’ management (i.e., TG-AAbs, see Section 4.2). Given that the TG-AAbs are also detected in a significant proportion of cancer-free individuals, TG cannot be regarded as a *bona fide* TAA. However, such a follow-up strategy provides a solid clinically validated proof-of-principle for the use of autoantibody biomarkers in thyroid cancer management.

### 5.1. TAAs Identified Using the Whole-Transcriptome/Proteome–Level Screens

**Figure 2:**
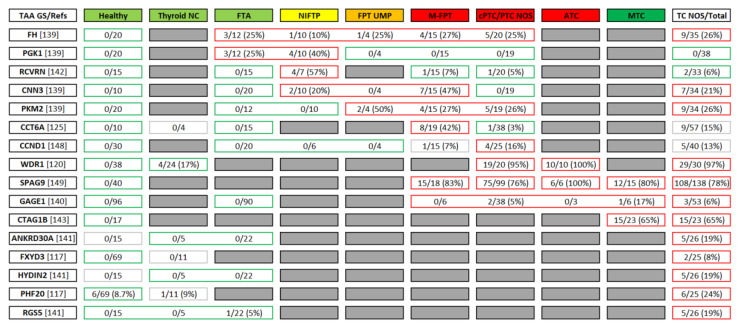
Summary of thyroid neoplasia-associated TAAs reported in the literature. Only those TAAs whose cognate TAA-AAbs demonstrate significant (*p* < 0.05 according to Fisher’s exact test) differences in frequencies between particular groups of individuals (contoured red for TAA-AAbs-high and green for TAA-AAbs-low groups), including relevant combined groups, are listed. Cells corresponding to groups absent from a particular study and to those that do not demonstrate significant differences in TAA-AAbs’ frequencies when compared with other groups in the study are colored and contoured grey, respectively. ATC—Anaplastic Thyroid Carcinoma; cPTC—Papillary Thyroid Carcinoma, classic/conventional type; FTA—Follicular Thyroid Adenoma; HCA—Hürthle Cell Adenoma; FPT—Follicular-Patterned Tumor; FPT-UMP—FPT of Uncertain Malignant Potential; GS—Gene Symbol; M-FPT—Malignant FPT (including Hürthle cell tumors); MTC—Medullary Thyroid Carcinoma; NIFTP—Non-Invasive Follicular thyroid Tumor with Papillary-like nuclear features; NOS—Not Otherwise Specified; TAA—Tumor-Associated Antigen; TC—Thyroid Cancer; Thyroid NC (non-clonal)—non-neoplastic thyroid diseases.

Using the classical top-down technique in the field, the Serological Analysis of Tumor Antigens by cDNA Expression Cloning (SEREX) [116], Kiyamova et al. [117] isolated a number of recombinant phage clones that were reactive with thyroid cancer sera with varying frequencies. In particular, autoantibodies recognizing a clone encoding an FXYD domain-containing ion transport regulator 3 FXYD3 were completely absent from the group of healthy controls and various benign conditions and demonstrated a significant difference in frequencies between groups of patients with thyroid cancer and healthy controls (Figure 2). Additionally, the frequency of TAA-AAbs recognizing clones corresponding to PHD finger protein 20 PHF20, also known as Glioma-Expressed Antigen 2 GLEA2 [118,119], was also significantly higher in thyroid cancer patients than in healthy controls, although these clones also demonstrated rare positive reactions in the latter group and in patients with benign thyroid lesions.

Using a similar approach, Izawa et al. [120] screened a phage cDNA expression library prepared from anaplastic thyroid carcinoma (ATC) tissue with an autologous serum to identify two immunogenic TAAs recognized by patient’s autoantibodies, represented by WD repeat domain 1 protein (WDR1) and fibronectin 1 (FN1). During the validation step of the study, WDR1-AAbs demonstrated excellent diagnostic performance in the discrimination of the combined PTC+ATC group vs. benign thyroid disease represented by FTA and various non-neoplastic lesions with ROC AUC 0.969 and DSn and DSp values of 97% and 92%, respectively, at the optimal cut-off level in the ELISA analysis (Figure 2). A second autoantigen FN1, although omitted from the validation step by the authors of this study, is also of particular interest. FN1 was implicated both in PTC pathogenesis and as a tissue-based biomarker of malignancy and poor prognosis [121,122,123]; the fact that this protein may also trigger the autoantibody response in thyroid cancer patients definitely warrants further studies in order to comprehensively address its diagnostic and prognostic potential.

In our own studies, we deliberately adhered to the translationally relevant setting of the most diagnostically challenging lesions, i.e., follicular-patterned tumors (FPT), the majority of these falling into the TBSRTC DC4 category (Figure 1-DN1, see Section 4.2). Using Serological Proteome Analysis (SERPA) [124], we were able to identify the T-complex protein 1 subunit ζ (TCP-1ζ, also known as chaperonin containing Tcp1 subunit 6A, CCT6A) as an autoantigen eliciting the autoantibody response in almost half of the patients with borderline and malignant encapsulated FPT, while being completely absent from patients with benign FTA (Figure 2). In TBSRTC DC4 nodules, CCT6A-AAbs were capable of detecting borderline and malignant tumors with DSp and DSn values of 100% and 40%, respectively [125]. Interestingly, more recent studies extensively implicated TCP-1ζ/CCT6A as an oncogenic protein and biomarker of poor prognosis in various human malignancies [126,127,128,129,130,131,132,133,134,135,136,137,138], rendering its cancer-associated immunogenicity highly biologically relevant.

Even more intriguingly, in some cases from our study, malignancy was unveiled as a result of a pathological reassessment of tumors in CCT6A-AAbs-positive patients [125]. Thus, such a biomarker may be useful not only for a workup of patients with indeterminate FNA cytology but also for the identification of nodules initially diagnosed as benign but actually representing a minimally invasive malignancy with subtle invasive foci residing away from the sectioned planes (Figure 1-DN3, see also Section 4.2).

We further elaborated our top-down research to more comprehensively study the TAAs’ repertoire of the well-differentiated follicular cell-derived thyroid tumors using an extended panel of samples both at the screening and validation steps, as well as the improved multi-dimensional proteomic approach allowing simultaneous analysis of the protein expression differences between NRAS^Q61R^-expressing and mock-transfected thyroid cells and serum autoantibody reactivity against these proteins [139]. Using this pipeline, we were able to expand the list of the autoantibody response-eliciting TAAs in follicular cell-derived thyroid tumors by four proteins, namely, glycolytic enzymes pyruvate kinase PKM2 [27,28,29,30,31,32] (see Section 3.1) and phosphoglycerate kinase PGK1, a thin filament-associated protein calponin-3 CNN3, and the tricarboxylic cycle enzyme fumarate hydratase FH.

Notably, three of them (PKM2, PGK1, and CNN3) demonstrated highly significant differences in the cognate TAA-AAbs’ frequencies between different histological types of tumors, and each of the cognate TAA-AAbs were capable of 100% specific partitioning of TAA-AAbs-positive cases in a particular group of histological phenotypes: overtly malignant (including classical PTC) + borderline tumors (excluding NIFTP) for PKM2-AAbs+ cases; overtly malignant + borderline FPT (including NIFTP) for CNN3-AAbs+ cases; and non-invasive tumors (FTA/HTA + NIFTP) for PGK1-AAbs+ cases with moderate (30-35%) DSn values (Figure 2). Upon limiting the analysis to FPT (i.e., tumors most frequently falling into TBSRTC DC3 and TBSRTC DC4 categories) and using the combined autoantibody score (AAS) calculated for a three-antigen panel, the pre-test risk of malignancy was successfully reclassified in almost half (46%, 19/41) of FPT patients [139].

### 5.2. TAAs Implicated in Thyroid Neoplasia in Bottom-Up Setting

Apart from TAAs identified in a few top-down studies carried out at a whole-transcriptome/proteome-level, several additional TAAs were also implicated in thyroid neoplasia either in bottom-up or in a «semi bottom-up» (i.e., selection of TAA candidates from a set of pre-selected protein features) settings. In such a way, Abols et al. [140] used a low-density phage-displayed TAA microarray comprising 65 candidate TAA features in order to test their serological reactivity in 53 patients with various thyroid carcinomas, 90 FTA patients, and 96 cancer-free individuals. Although the differences in frequencies of the cognate TAA-AAbs’ reactivity between studied groups reached statistical significance for only a single TAA (i.e., cancer-testis antigen GAGE1, Figure 2), the optimal cut-off values of combined serum score calculated for a six-antigen TAA panel preferentially reacted with thyroid cancer sera (i.e., GAGE1, COP9 signalosome complex subunit 4 (COPS4) and four artificial peptides most likely encoding the mimotopes of unknown antigens) discriminated the combined thyroid cancer group from FTA and healthy individuals with DSp of 97% and 90%, respectively, and DSn of 25% in both comparisons.

In a somewhat similar study, we used a mini-array of 21 recombinant TAAs identified in non-thyroid human cancers to test their serological reactivity in 26 DTC and 22 FTA patients [141]. We were able to ultimately identify a three-features TAA signature (ankyrin repeat domain containing-protein 30A ANKRD30A, also known as NY-BR-1; regulator of G protein signaling 5 RGS5; and a particular isoform of hydrocephalus-inducing 2 HYDIN2 - KIAA1864) capable of significantly discriminating DTC from FTA cases both using individual TAAs (DSn of 19% for each; DSP of 95–100%) (Figure 2) and combined three-features TAA panel (DSn of 50%; DSp of 95%). Additionally, a TAA from the initial panel (a cancer-retina antigen recoverin RCVRN) was subsequently demonstrated to elicit a frequent TAA-AAbs response specifically in patients with NIFTP, significantly discriminating them from classical PTC, FTA, invasive FV-PTC, and healthy volunteers with DSp values of 93–100% and DSn values of 57% (Figure 2) [142].

In yet another study, Maio et al. [143] analyzed the expression of 11 cancer-testis antigens’ mRNAs in 23 surgical specimens of sporadic medullary thyroid carcinoma (MTC). The cognate TAA-AAbs reactivity against the antigen CTAG1B/NY-ESO-1, whose mRNA was most frequently expressed in MTC tissues (15/23 tumors, 65%), was also analyzed and revealed the presence of CTAG1B-AAbs in 15/42 of MTC patients (DSn 36%) but not in a single healthy control, thus yielding DSp values of 100% in the discrimination of MTC patients from the cancer-free individuals (Figure 2). Due to the rarity of MTC among the general population of patients with thyroid nodules, I did not address the current problems of MTC diagnosis in this review; nevertheless, such biomarkers may be useful in the context of moderately elevated calcitonin values to reliably distinguish patients with MTC from those with benign C-cell hyperplasia, the problem that is still far from being completely resolved (reviewed in [144]).

Finally, several TAAs were implicated in thyroid neoplasia in a fully bottom-up setting. In such a way, we hypothesized that cyclin D1 CCND1, a critical positive regulator of the cell cycle that was extensively implicated in the pathogenesis and tissue-based diagnostics of PTC (reviewed in [145]) but was also described as a target of TAA-AAbs in other cancers [146,147], may also trigger the diagnostically relevant autoantibody response in thyroid tumors. The CCND1-AAbs were ultimately found in 4/25 (16%) of patients with classical PTC but were completely absent from all classes of FPT and healthy controls (Figure 2) [148]. In another study, Garg et al. [149] demonstrated exceptionally frequent (76–100%) TAA-AAbs response against a cancer-testis antigen sperm-associated antigen 9 SPAG9 across the whole spectrum of malignant thyroid neoplasms (Figure 2). However, given that the authors did not address the expression and cognate TAA-AAbs reactivity of SPAG9 in benign thyroid lesions, the real-life diagnostic potential (i.e., DSp) of such a sensitive biomarker is yet to be elucidated.

## 6. The Conceptual Problems in the Identification of TAAs in Thyroid Neoplasia

As discussed in Section 4.2, current diagnostic strategies in the management of patients with NTD and DTC have many windows for improvement (Figure 1). The extensive research on TAA-AAbs conducted in other types of human cancer (see Section 3) provides strong rationales for the potential use of TAA-AAbs in similar clinical contexts in thyroid oncology, whereas studies on thyroid neoplasia–associated TAAs and TAA-AAbs (Figure 2), albeit not that numerous, nonetheless provide a solid proof-of-principle for the diagnostic use of TAA-AAbs in patients with NTD (see Section 5). However, to proceed to the clinically applicable diagnostic tools, many points are yet to be addressed both with reference to already identified TAAs and TAA-AAbs and to the future top-down studies in the field.

With respect to TAAs and TAA-AAbs already identified in thyroid neoplasia (Figure 2, see also Section 5), their diagnostic performance and clinical applicability should be tested in well-designed prospective studies conforming to the real-life diagnostic demands (non-exhaustive representatives of the latter being discussed in Section 4.2 and depicted in Figure 1). Given that the differential diagnosis in patients with TBSRTC DC3 and TBSRTC DC4 nodules probably represents the most critical issue in the field (see Section 4.2), the capability of identified TAA-AAbs to significantly discriminate between malignant FPT and benign follicular lesions (i.e., benign FPT and non-clonal proliferations) is the key to success. Hence, many candidate TAA-AAbs initially identified in the discovery setting (e.g., comparison of a non-stratified group of thyroid cancer patients vs. cancer-free controls) may ultimately turn out to be inapplicable in this clinical context.

For example, TAA-AAbs against fumarate hydratase FH (FH-AAbs) frequently occur in malignant thyroid tumors while being absent from cancer-free controls; however, benign and malignant thyroid neoplasms demonstrate quite comparable frequencies of FH-TAAbs (Figure 2), thus precluding their use in any setting that requires discrimination between benign and malignant entities. In another scenario, an antigen initially identified as a target of TAA-AAbs in a non-stratified cohort of thyroid cancer patients and significantly discriminating them from cancer-free controls and even benign tumors (e.g., cyclin D1 CCND1) may again fail to add any diagnostic information in a real-life setting due to its preferential autoantibody reactivity in classical type PTC (Figure 2), a tumor type representing the majority of thyroid cancer in general (and thus being highly enriched in non-stratified thyroid cancer groups) but being usually easily diagnosed using FNA cytology (TBSRTC DC5 and TBSRTC DC6) and thus being only occasionally encountered among TBSRTC DC3 and TBSRTC DC4 nodules.

The same considerations are fully applicable not only to validation studies but also to the study design in TAAs and TAA-AAbs discovery research. Moreover, improper balancing of specific pathological entities’ prevalence in the discovery setting may yield not only the enrichment of the resultant signatures with diagnostically irrelevant TAAs but also the failure of an experimental protocol to identify a subset of highly diagnostically significant TAAs whose serological reactivity is restricted to the most diagnostically important albeit grossly relatively rare entities (e.g., CCT6A-AAbs in malignant FPT, Figure 2).

Another interesting point related to the considerations above pertains to the differential diagnosis in TBSRTC DC3 and TBSRTC DC4 nodules (and, in particularly, between the FPT of different malignant potential) is the direction of association between the presence of TAA-AAbs of the particular specificities and malignant potential of a tumor. In many studies on TAA-AAbs (including those discussed in Section 5), the discovery step of a study (e.g., primary SEREX screening, high-density microarray profiling of TAA-AAbs’ repertoire in a small discovery sample, etc.) would only include cancer patients and cancer-free controls, with various benign conditions being further tested in a validation step of a study once a primary list of candidate TAAs is established. In such a setting, only cancer-specific TAAs and TAAs with a somewhat promiscuous pattern of serological reactivity (e.g., FH) are to be identified. In contrast, those TAA-AAbs that may be absent from cancer patients but specifically encountered in patients with benign neoplasms will be completely missed at the very initial step of a study.

As discussed in Section 2 and explicitly illustrated throughout the present review, TAA-AAbs usually demonstrate very high DSp but low-to-moderate DSn values, i.e., the TAA-AAbs—positivity would reliably partition a case in a particular clinically relevant group (e.g., patients with cancer), while the absence of TAA-AAbs would not substantially modify the pre-test risk of having a condition of interest. This point is also clearly evident from published data on thyroid neoplasia-associated TAAs (Figure 2, see also Section 5). The benign tumor-associated TAA-AAbs absent from patients with cancer are thus expected to confidently rule out malignancy in a somewhat implicit way (i.e., via ruling in a benign condition). For example, autoantibodies against a SEREX-defined TAA Sry-HMG-box 2 SOX2 are detected in 23% of patients with monoclonal gammopathy of undetermined significance (MGUS), a benign condition representing a precursor lesion to multiple myeloma (MM), but are completely absent from patients with MM (i.e., DSp for MGUS 100%) [150]. Similarly, TAA-AAbs against the meningioma-expressed antigen 11 MGEA11 are encountered in 50% of patients with the World Health Organization (WHO) grade I benign meningioma but are completely absent from patients with atypical (WHO grade II) and anaplastic (WHO grade III) meningiomas [151]. In such cases, the presence of TAA-AAbs of a particular specificity would confidently rule out malignant disease and, even more importantly, may represent a surrogate marker of the efficient immune control over the benign neoplastic lesion halting its progression to frank malignancy [150].

The degree to which these considerations may be applied to thyroid neoplasia is yet to be determined, although our proof-of-principle data indicate that inclusion of benign tumor sera in a screening sample may actually result in the successful identification of such benign or indolent borderline tumor-associated TAAs (i.e., PGK1 or RCVRN, Figure 2). Thus, further studies specifically focusing on the identification of such TAAs are definitely warranted, given that their inclusion in diagnostic panels would be capable of significant expansion of functionality of the latter via confident downgrading of a pre-test risk of malignancy [150], an option that is barely achievable using TAAs with a cancer-specific pattern of serological reactivity.

## 7. Perspectives and Conclusions

Apart from the issues discussed in detail in previous sections (Figure 1-DN1-DN6), one may suggest further use of TAA-AAbs in additional clinical contexts in thyroid neoplasia. These clinical niches include the prediction of the tumor response to ^131^I RAI therapy (Figure 1-DN8), further assessment of the risk of malignancy in patients with TI-RADS 3-6 nodules who are scheduled for a follow-up due to a small nodule size (Figure 1-DN9), and the whole spectrum of diagnostic implications in rare or non-follicular cell-derived thyroid tumors (e.g., MTC, see Section 5.2). Whereas data allowing TAA-AAbs in these contexts to be reasonably implicated is rather sparse, yet the use of these modalities may be advocated based on theoretical considerations and data obtained in non-thyroid human cancers. For example, the efficacy of ^131^I RAI ablation is not uniform in all subtypes of DTC, and accurate prediction of ^131^I RAI efficacy both prior to the treatment and following ^131^I administration may represent a relevant diagnostic niche for TAA-AAbs (Figure 1-DN8), particularly given that ^131^I RAI ablation may appear to be a strong immunogenic stimulus due to the massive release of intracellular antigens upon the dying of normal thyroid and DTC cells. The degree to which such an effect may be associated with the fueling of the anti-tumor immunity (including the production of TAA-AAbs), the possibility of synergy between cytotoxic ^131^I RAI therapy and the anti-tumor immune response, as well as prognostic implications thereof, are very interesting questions to be addressed in future studies. The well-designed top-down studies aimed at a comprehensive survey of repertoires of TAA-AAbs’ specificities in well-established and emerging clinical contexts pertaining to the management of patients with NTD and DTC would be of great translational value in endocrine practice, and their need is strongly advocated by unoccupied diagnostic niches in the management of patients with NTD, well-established implications of TAA-AAbs in similar contexts in other human cancers, as well as by proof-of-principle studies demonstrating a substantial diagnostic potential of TAA-AAbs in thyroid oncology.
